# Malignant external otitis complicated by spinal cord compression: Case report

**DOI:** 10.1016/j.amsu.2022.104775

**Published:** 2022-09-24

**Authors:** Hiba Safi Eddine, Kenza Gourram, A. Merzem, N. Moussali, Nel Benna, Youssef Bouzoubaa, Loubna Douimi, S. Rouadi, R. Abada, M. Mahtar

**Affiliations:** a Radiology Department, University Hospital Center 20 Aout 1953, Casablanca, Morocco; b Otorhinolaryngology Department, University Hospital Cente 20 Aout 1953, Casablanca, Morocco; c Faculty of Medicine and Pharmacy, Hassan II University, Casablanca, Morocco

## Abstract

**Introduction:**

Malignant otitis externa is a life-threatening infectious pathology that occurs mainly in diabetic patients; in a picture of otorrhea, with facial paralysis. We report the case of a necrotizing otitis externa, treated with antibiotics, which was complicated a few months later by spinal compression.

**Observation:**

This is the case of a 60-year-old patient, diabetic, who presented a painful otorrhea associated to left facial palsy.

The diagnosis of necrotizing otitis externa was retained after performing a CT scan of the temporal bone.

6 months after medical treatment, the patient became has been complicated by tetraparesis with respiratory distress.

**Discussion:**

Necrotizing otitis externa is an osteitis of the base of the skull, which occurs in the diabetic patient and which starts in the external ear and spreads by contiguity after infection of the temporal bone.

It represented clinically by otalgia and purulent otorrhea.

The role of imaging is to confirm the involvement and to specify the extension of the lesions. CT scan is useful to evaluate the bone involvement. MRI is the examination of choice for the study of soft tissues and is essential in advanced forms.

Its treatment is based on antibiotic therapy for a minimum of 6 weeks.

**Conclusion:**

OEM is an infection occurring in elderly and diabetic patients. Imaging allows to confirm the diagnosis and to carry out the assessment of extension; but also has a great interest in the follow-up of these immunocompromised patients who are subject to complications with insidious evolution

## Highlights

• Identify high risk patients of malignant otitis externa.
• Review the appropriate evaluation of this pathology and its complications.
• Describe interprofessional team strategies of surveillance for improving care coordination.

## 1 Introduction

Malignant otitis externa or skull base osteomyelitis is an invasive infectious pathology involving the vital prognosis; which diffuses according to a mode of osteitis with bone destruction and can simulate an expansive pathology. It occurs mainly in diabetic patients; in a picture of otorrhea, with facial paralysis. We report the case of a necrotizing otitis externa, initially treated by double antibiotic therapy (C3G + Fluoroquinolones) by injection for “weeks” and then switched to the oral route for other weeks, complicated several months later by medullary compression.

This observation shows the interest of radiological surveillance in these immunocompromised patients who can be prone to reccurrences with atypical clinical signs responsible for diagnostic delays

This work has been reported with respect to the SCARE 2020 criteria [1].

## 2 Observation

We report on this observation, the case of a 60 years old patient, diabetic under oral antidiabetics, who initially presented a painful otorrhea associated with a facial paralysis of the left side, with progressive evolution.

On clinical examination: He was presenting with a red, edematous external ear with pus discharge.

A bacteriological sample allowed the isolation of Pseudomonas Aeruginosa. The histological study came back non specific: inflammatory polyp.

**Fig. 1:**
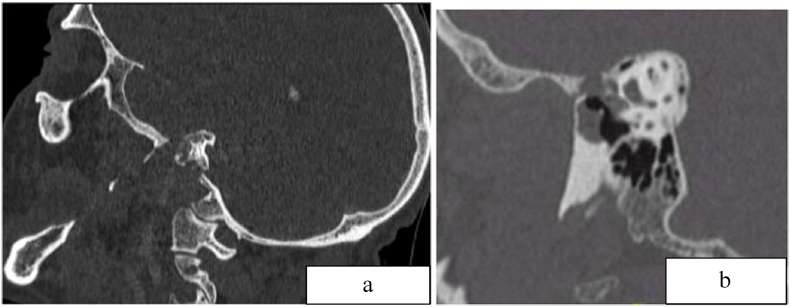
a, b: Process occupying the tympanic cavity and external auditory canal responsible of temporal lysis and enlargement of the jugular formana

A CT scan of the temporal bone revealed an aggressive lytic process centered on the jugular foramen and extended to the deep spaces of the face responsible for thrombosis of the internal jugular vein and the homolateral sigmoid sinus, with filling of the tympanic cavity and partial lysis of the ossicular chain (Fig. 1). The diagnosis of necrotizing otitis externa was retained in view of these signs.

The patient was treated with C3G and fluoroquinolones during 6 weeks, combined with topical preparations and local care. The CT scan after 1 month showed an almost stable aspect of the lytic process despite the favorable clinical evolution.

**Fig. 2:**
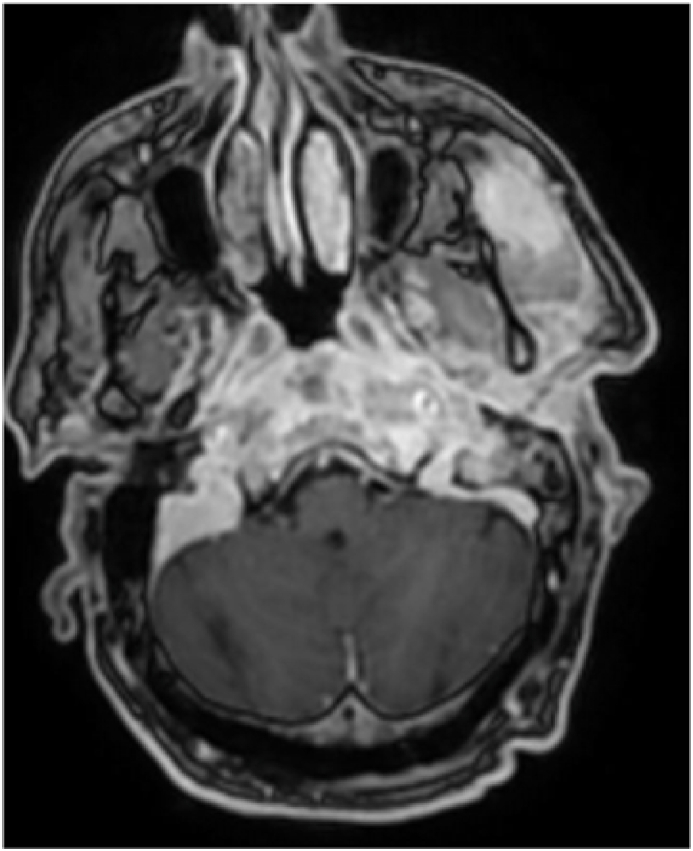
Cervical MRI showing infiltration and enhancement of the inner ear extended to the deep spaces of the face related to malignant otitis externa.

**Fig. 3:**
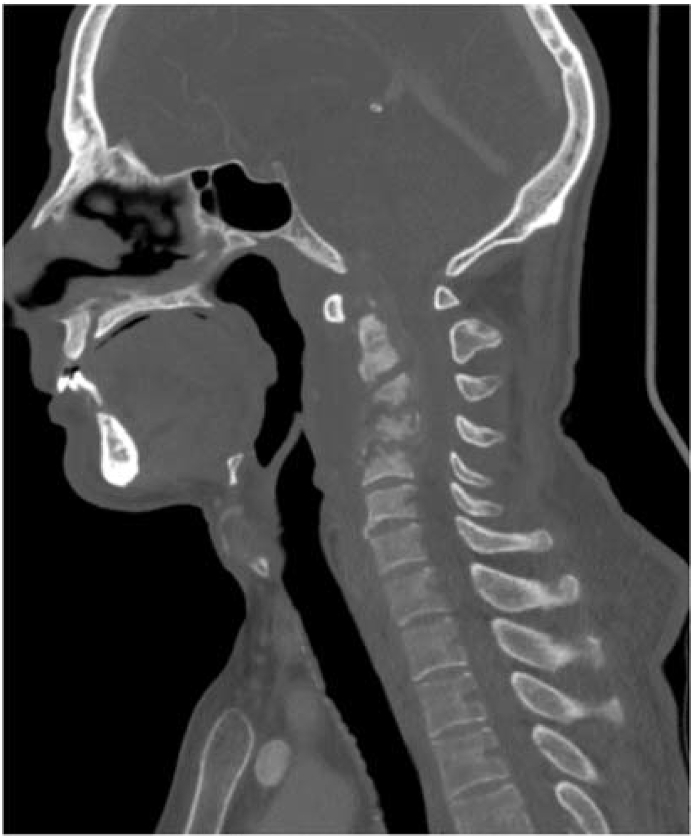
Vertebral lysis due to spondylodiscitis.

**Fig. 4:**
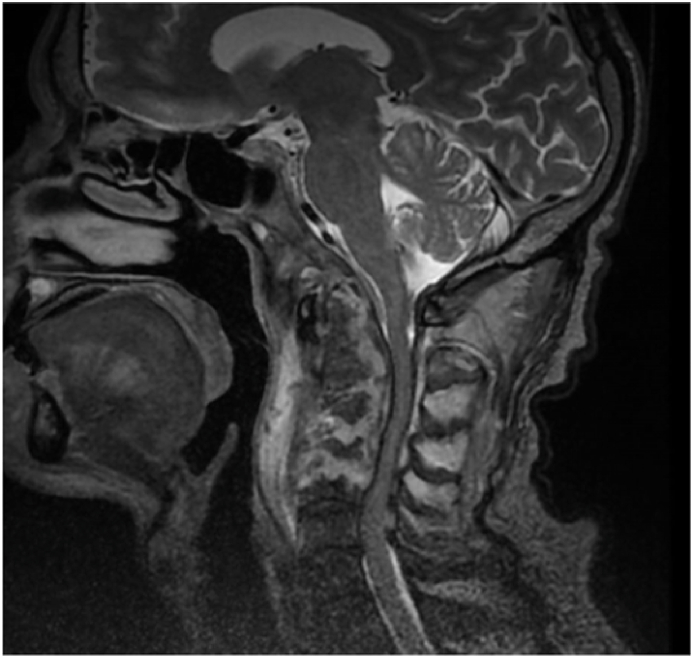
Cervical spondylodiscitis complicated by prevertebral and epidural abscesses.

After 6 months, the patient developed tetraparesis with respiratory distress for which he was hospitalized in the intensive care unit. In view of the clinical picture, a cervical MRI was performed which revealed a thickening of the prevertebral soft tissues extending from the nasopharyngeal level, opposite the clivus, up to C6 infiltrating the vertebral bodies with intra dural extension (Fig. 2). This thickening was responsible of a significant mass effect on the marrow opposite. There was an associated contrast of the inner and middle ear and the external auditory canal without intracranial extension. On complementary CT scan, to evaluate the extent of the bone involvement, we found lysis of the vertebral bodies, the ossicular chain, and the walls of the EAC (see Fig. 3, Fig. 4).

The patient died 48 hours after admission due to cardiorespiratory arrest.

## 3 Discussion

Malignant otitis externa is an osteitis of the skull base, which starts in the external auditory canal and spreads by contiguity after infection of the temporal bone. It is a rare but serious complication of otitis externa, which can be life-threatening because of the extension of the infection to the deep spaces of the face and the central nervous system. The risk factors are diabetes (poor tissue perfusion by micro-angiopathy and the higher pH of the external auditory canal that it causes), age (probably related to immuno-senescence and vascular disorders responsible for tissue alteration by ischemia), immunodepression and local trauma to the external auditory canal (washing, cotton swab, hearing aid …) responsible for an effraction of the cerumen-epithelial barrier [2].

Clinically, it presents with a subtle, trivial otalgia, which explains the diagnosis delay, but which gradually increases, associated to a classically purulent otorrhea. Hypoacusis is inconstant and moderate, vertigo and trismus are rarely found.

Facial paralysis, often complete, is present in 20–50% of cases depending on the series. Other cranial nerves may also be affected. This nerve damage is a sign of extension to the skull base or, more rarely, to the cavernous sinuses [3].

As for imaging, its role is to confirm the damage and to specify the extension of the lesions which is generally towards the temporo-mandibular joint in front, towards the mastoid in the back; the petrous apex inwards and the cervical spaces in depth. The CT scan is useful to evaluate the bone involvement which starts 3–5 days after the onset of the infection by generally showing osteolysis of the bone cortex associated to a swelling of the soft parts of the EAC [4].

MRI is the preferred modality for the study of soft tissues and is essential in advanced forms. However, these modalities do not allow the differential diagnosis with tumor processes and therefore a lack of specificity. The combination of radiological and radionuclear examinations is becoming increasingly important for the initial diagnosis and follow-up of patients [5].

Its treatment is based on intravenous antibiotic therapy for 6 weeks combined with topical preparations and local care. Surgery is no longer necessary except in cases that are resistant to medical treatment and should be limited to simple debridements.

But despite the improvement of the vital prognosis, the recurrences are still frequent in this pathology and vary from 10 to 25% depending on the series.

These recurrences are characterized by the resumption of otalgia and sometimes of otorrhe a re-ascension of the sedimentation rate.

They can occur up to twelve months after the antibiotics have been stopped which requires regular and prolonged follow-up.

These recurrences can sometimes be massive, with involvement of the skull base [6] and given the paucity and atypicity of clinical signs in immunocompromised patients, imaging surveillance remains the most reliable means of ensuring complete resolution

## 4 Conclusion

Malignant otitis externa is a serious infection, its management is a real challenge for the clinician. Occurring in elderly and diabetic patients, its evolution is very variable, ranging from a simple superficial infection to endocranial damage with a poor prognosis.

Its treatment is based on prolonged parenteral antibiotic therapy.

Imaging is used to confirm the diagnosis and to carry out an extension assessment. A CT scan of the temporal bone is the first-line examination to look for bony extension, supplemented by an MRI scan to analyze the soft tissues and the deep cervical spaces and to look for signs of endocranial extension and also has a great interest in the follow-up of these immunocompromised patients who are subject to complications with insidious evolution.

## Conflicts of interest

The authors declare having no conflicts of interest for this article.

## Funding

None.

## Ethical approval

I declare on my honor that the ethical approval has been exempted by my establishment.

## Consent

Written informed consent for publication of their clinical details and/or clinical images was obtained from the patient.

## Author contribution

Safi Eddine Hibatou ALLAH: Corresponding author writing the paper.

## Registration of research studies

None.

## Guarantor

Dr SAFI EDDINE HIBATOU ALLAH.
